# PlantMirP2: An Accurate, Fast and Easy-To-Use Program for Plant Pre-miRNA and miRNA Prediction

**DOI:** 10.3390/genes12081280

**Published:** 2021-08-21

**Authors:** Dashuai Fan, Yuangen Yao, Ming Yi

**Affiliations:** 1 School of Mathematics and Physics, China University of Geosciences, Wuhan 430074, China; fandashuai@cug.edu.cn; 2 Department of Physics, College of Science, Huazhong Agricultural University, Wuhan 430070, China; yyg@mail.hzau.edu.cn

**Keywords:** microRNA, pre-miRNA, support vector machine, knowledge-based energy feature

## Abstract

MicroRNAs (miRNAs) are a kind of short non-coding ribonucleic acid molecules that can regulate gene expression. The computational identification of plant miRNAs is of great significance to understanding biological functions. In our previous studies, we have put firstly forward and further developed a set of knowledge-based energy features to construct two plant pre-miRNA prediction tools (plantMirP and riceMirP). However, these two tools cannot be used for miRNA prediction from NGS (Next-Generation Sequencing) data. In addition, for further improving the prediction performance and accessibility, plantMirP2 has been developed. Based on the latest dataset, plantMirP2 achieves a promising performance: 0.9968 (Area Under Curve, AUC), 0.9754 (accuracy), 0.9675 (sensitivity) and 0.9876 (specificity). Additionally, the comparisons with other plant pre-miRNA tools show that plantMirP2 performs better. Finally, the webserver and stand-alone version of plantMirP2 are available.

## 1. Introduction

MicroRNAs (miRNAs) are small noncoding RNAs with a length of about 20–24 nucleotides [1]. Plant miRNAs play important functions in plant growth, development and responses to abiotic and biotic stresses [2]. For example, MdmiR285N microRNA is involved in the biotic stress response, plant growth and reproductive development in apple (*Malus* × *domestica*) and *Arabidopsis thaliana* [3]. The miR396–GRF/GIF system can effectively regulate plant growth, and is a promising target for increasing plant yield [4]. Under drought conditions, the miR156/157 and miR399 families can regulate the expression of transcription factor to enhance the viability of plants [5].

Accurate detection and identification of plant miRNAs are the basis of understanding miRNA biological functions. Therefore, many methods have been developed to this area. These methods are roughly divided into two categories: One is based on biological experiments [6,7,8,9], and the other is based on computational prediction. Traditional experimental methods are usually time-consuming, laborious and inefficient, and may even miss miRNAs with low expression levels. Computational methods can make up for these shortages of traditional experimental methods, and thus attract more and more attention. Computational methods for miRNA identification can be classified into the following categories: Homology comparison-based [10,11], high-throughput sequencing-based [12,13] and machine learning-based methods. Machine learning-based methods are the most popular microRNA prediction methods and have shown superior performance.

In 2005, Xue et al. used 32 local structural features to build an SVM (Support Vector Machine) model (triplet-SVM) for predicting human pre-miRNA [14]. In 2007, based on the same data source with triplet-SVM, Ng et al. extracted 29 features and constructed the classifier miPred [15]. Based on miPred, Batuwita et al. constructed 19 structure-related novel features, and optimized the negative sample set to train the classifier microPred [16]. These methods are specifically designed to predict animal pre-miRNAs rather than plant pre-miRNAs. In fact, the number of tools for specifically predicting plant pre-miRNA is relatively few. One possible reason is that the secondary structure of plant pre-miRNAs is more complex than that of animals so that plant pre-miRNAs are more difficult to be predicted. In 2011, Ping et al. constructed the an SVM-based PlantMiRNAPred for plant pre-miRNA [17]. In addition, there are some typical tools used specifically for plant pre-miRNA detection, such as random forest-based HuntMi [18], SVM-based miPlantPreMat [19] and semi-supervised learning-based MiRNAss [20]. Especially, we have constructed a set of novel knowledge-based energy features that combine the *k*-mer scheme with knowledge-based potentials derived from Boltzmann formulations, and developed SVM-based plantMirP for predicting plant pre-miRNAs [21]. Additionally, plantMirP performs superiorly to existing tools at that time. However, the above-mentioned methods are tools for predicting pre-miRNA rather that miRNA. More importantly, these tools cannot directly deal with NGS (Next-Generation Sequencing) data from small RNA sequencing.

In 2012, based on a probability model, Friedlaender et al. exploited a probability model to comprehensively score the coincidence degree between sequencing fragments and microRNA production process, and proposed miRDeep [12] to directly predict miRNAs from NGS data. After that, some miRDeep-based tools came out, such as miRDeep2 (the updated version of miRDeep) [13], miRDeep* [22], miRDeep-P [23] and miRDeep-P2 (the updated version of miRDeep-P) [24]. Note that miRDeep-P [23] is the first computational tool for specifically retrieving plant miRNAs from sequencing data. In 2018, by introducing new plant miRNA annotation standards, Zheng et al. improved the strategies and algorithms of miRDeep-P, and proposed miRDeep-P2, which shows higher accuracy and is less time-consuming. Additionally, there are also some other methods, such as MIReNA [25], miRPlant [26], miR-PERFeR [27] and miRA [28].

These high-throughput sequencing-based miRNA prediction tools share a familiar flowchart. Firstly, sequence alignment tools are used to align sequencing fragments (i.e., reads) to reference genomes. Then, candidate regions are selected and further labeled as known microRNA precursors according to genome annotation data or nominated precursors according to sequence and structure-related characteristics. Based on the model of miRNA biogenesis, the compatibility of the position and frequency of the aligned reads with the microRNA precursors is evaluated in virtue of various different methods. These tools are not taking full advantage of sequence and structure features of miRNA precursors. Moreover, high-throughput sequencing-based methods usually predict superfluous miRNAs. However, the follow-up researchers usually select a few miRNAs to conduct verification of biological functions. Therefore, how to pick these miRNAs is a practical and thorny problem. In addition, those predicted miRNAs are not all genuine. How to control false positive rate is an important problem. We argue that the combination of high-throughput sequencing-based (which is based mainly on model of miRNA biogenesis) with machine learning-based methods (which is based on local structure-sequence features) will further boost the performance of miRNA prediction, narrow the range of selection and reduce the false-positive rate.

To do this, we developed plantMirP into plantMirP2. Firstly, we incorporated and optimized knowledge-based energy features, which are firstly proposed in plantMirP and further developed in our recent studies (i.e., riceMirP [29] and milRNApredictor [30]). Secondly, the parameters of the SVM model and the algorithm are optimized, and the independent dataset is updated according to the latest version of the miRBase database. The performance of plantMirP2 is obviously improved. Meanwhile, plantMirP2 runs significantly faster than the previous version. Based on 10-fold cross-validation (CV), plantMirP2 exhibits a promising performance: An accuracy of 97.55%, a sensitivity of 95.22%, a specificity of 99.00%, Mathew’s correlation coefficient of 0.9482 and an area under receiver operating characteristic curve (AUC) of 0.9930. This tool performs superiorly to existing plant pre-miRNA prediction tools. Then, we combined machine learning-based methods with high-throughput sequencing-based methods to further improve prediction performance, narrow the range of selection and reduce the false-positive rate. Finally, for the convenience of users, plantMirP2 is available as a stand-alone package (https://github.com/wuqiansibai/plantMiRP2/releases/tag/v1.0/, accessed on 16 August 2021) and a Dockerfile (https://github.com/wuqiansibai/plantMiRP2/releases/tag/Dockerfile/, accessed on 16 August 2021) alongside the web-server version (http://plantmi.top/, accessed on 16 August 2021), which is able to directly receive and process NGS data.

## 2. Materials and Methods

### 2.1. Datasets and Feature Set

Plant pre-miRNA sequence data (positive dataset) used in plantMirP was extracted from the miRBase [31] database (release 21), and now plant pre-miRNA sequence data used in plantMirP2 is extracted from the miRBase database (release 22.1). The method and standard, which are used here to generate positive and negative datasets, are the same as the old version. To be specific, after removing sequences containing non-AUCG characters, 3223 pre-miRNAs from 9 major plants (*Arabidopsis thaliana*, *Glycine max*, *Oryza sativa*, *Physcomitrella patens*, *Medicago Truncatula*, *Sorghum bicolor*, *Arabidopsis lyrata*, *Zea mays* and *Solanum lycopersicum*) were used as the positive training dataset, while 5323 pre-miRNAs from the remaining plants were used as the positive testing dataset. On the other hand, pseudo pre-miRNA fragments were extracted from CDS (Coding Sequence) data under the constraint that there are sufficient similarities between pseudo and genuine pre-miRNAs. Then, 8652 pseudo pre-miRNAs were randomly selected as the negative dataset including 5186 negative training dataset and 3466 negative testing dataset. The differences in positive datasets of both tools are shown in Table 1.

In plantMirP, we firstly designed knowledge-based energy features by combining the *k*-mer scheme in bioinformatics and the distance-specific pair potential in statistical physics. The recognition characteristic of *k*-mer and the advantages of distance-specific pair potential distinguishing between natural and non-natural structures were well combined. Furthermore, knowledge-based energy features have been firmly demonstrated to have very high discriminatory power [21,29,30]. Most recently, knowledge-based energy features have been further optimized and developed to consider position-specific information. All 193 features used in plantMirP2 are listed in Table 2.

### 2.2. Performance Evaluation

The AUC value under the ROC (Receiver Operating Characteristic) curve is a global measure for evaluating classification performance. A larger AUC value means a better classification performance. Accuracy (*Ac*), sensitivity (*Se*), specificity (*Sp*) and Matthew’s correlation coefficient (*MCC*) are widely used in the binary classifier. Their definitions are defined as follows:

(1) $$Ac=\frac{TP+TN}{TP+FP+TN+FN}$$

(2) $$Se=\frac{TP}{TP+FN}$$

(3) $$Sp=\frac{TN}{TN+FP}$$

(4) $$MCC=\frac{\left(TP\times TN\right)-\left(FN\times FP\right)}{\sqrt{\left(TP+FN\right)\times \left(TN+FP\right)\times \left(TP+FP\right)\times \left(TN+FN\right)}}$$

Here *TP* (True Positive) and *FP* (False Positive) represent the number of real positives and false positives, respectively, while *TN* (True Negative) and *FN* (False Negative) indicate the number of true negatives and false negatives.

### 2.3. SVMs

Here we use the algorithms associated with the sklearn package [32] in python to build the SVM model with kernel function:

(5) $$K\left(X_{i},X_{j}\right)=e^{-\gamma |X_{i}-X_{j}{|}^{2}}$$

and

(6) $$\gamma =\frac{1}{n\times \sigma^{2}}$$

where n is the number of features and $\sigma^{2}$ is the generalized variance. Then, through the grid search strategy, the parameters of the kernel function and the penalty parameter *C* (which is used to reduce the degree of overfitting) are adjusted based on the results of cross-validation.

When training the SVM model, the input is the 193 feature values of training sequences, and these feature values are normalized using the following formula:

(7) $$x^{*}=\frac{x-\mu }{\sigma }$$

The training scalers are saved for processing the feature values of testing sequences. During training the SVM model, the provided target variable y is true (1) or false (0) and this is also the target output during testing.

### 2.4. Implementation of PlantMirP2 Stand-Alone and Web-Server

**Figure 1:**
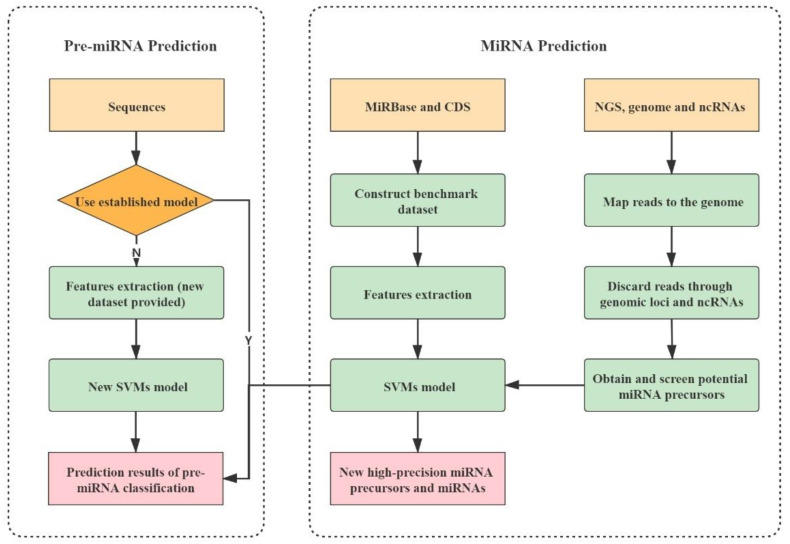
Flowchart of plantMirP2.

**Figure 2:**
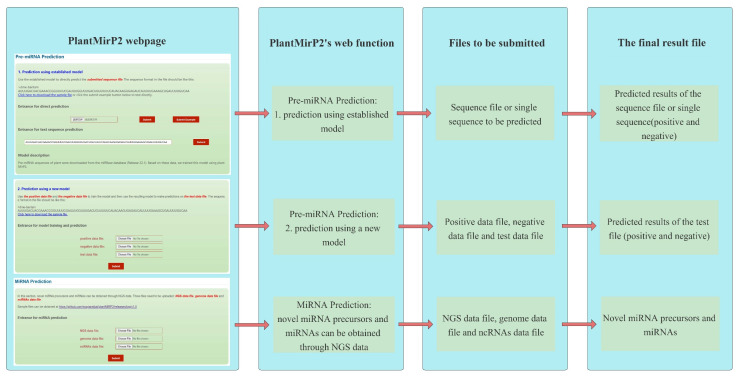
Webpage description and function introduction of plantMirP2′s webserver.

PlantMirP2 was constructed in python and Perl according to the flowchart (Figure 1). Fundamental packages from the python and perl library were also used. More details can be seen in the links below. The local package of plantMirP2 is provided at https://github.com/wuqiansibai/plantMiRP2/releases/tag/v1.0/ (accessed on 16 August 2021) and the related Dockerfile is provided at https://github.com/wuqiansibai/plantMiRP2/releases/tag/Dockerfile/ (accessed on 16 August 2021). All scripts have been tested on CentOS. For the convenience of users, we also provide the webserver of plantMirP2 (http://plantmi.top/, accessed on 16 August 2021) for pre-miRNA prediction, and direct miRNA prediction (Figure 2) from NGS data based on miRDeep-P2 [24], which used an a priori probability model specifically designed to overcome more variations in the length of pre-miRNAs and problems of more prevalent large paralogous families in plant NGS data.

## 3. Results

### 3.1. An Improved Algorithm for the Prediction of Plant Pre-miRNAs

**Figure 3:**
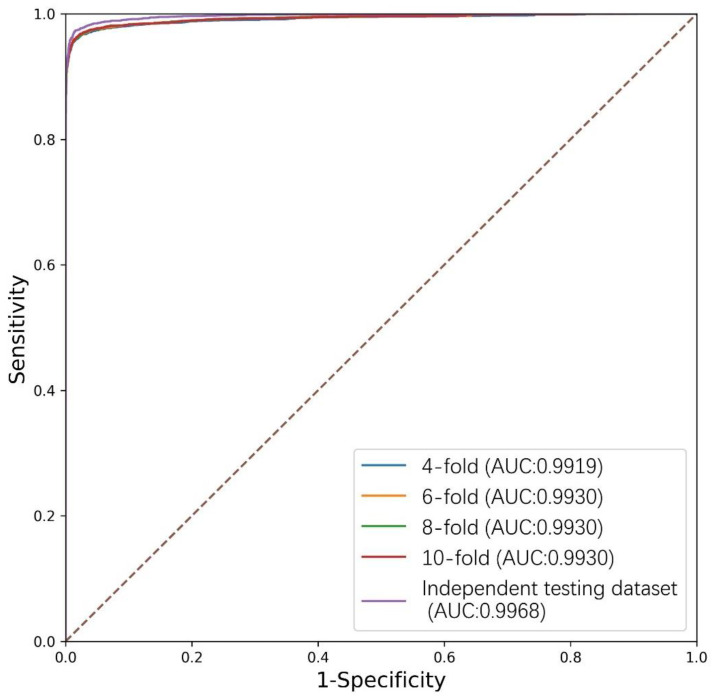
ROC (Receiver Operating Characteristic) curves and corresponding AUC (Area Under Curve) values of the 4-, 6-, 8- and 10-fold CVs (Cross-Validations) based on the training dataset and the independent testing dataset.

**Figure 4:**
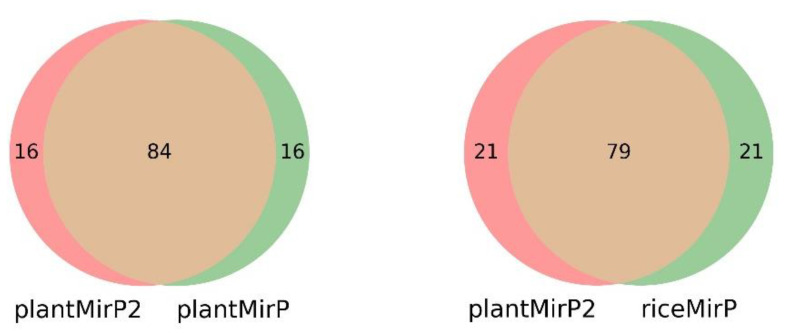
Venn diagram results for the top predictions of plantMirP2, plantMirP and riceMirP.

PlantMirP2 models were trained using 3044 positive training data and 5186 negative training data and tested using 3865 positive testing data and 3466 negative testing data. To evaluate the performance and robustness of the plantMirP2, 4-, 6-, 8- and 10-fold CVs were performed based on the training dataset. The AUC values under the ROC curve are 0.9919 (4-fold CVs), 0.9930 (6-fold CVs), 0.9930 (8-fold CVs) and 0.9930 (10-fold CVs), respectively (Figure 3). The ROC curves of 4-, 6-, 8- and 10-fold CVs are very close to each other, indicating that plantMirP2 is very robust. The values of AUC, Ac, Se, Sp and MCC of 10-fold CVs are 0.9930, 0.9755, 0.9522, 0.9900 and 0.9482, respectively. On the other hand, based on the independent (unseen) testing dataset, plantMirP2 also performs excellently and the values of AUC, Ac, Se, Sp and MCC are 0.9968, 0.9754, 0.9675, 0.9876 and 0.9493, respectively. Considering the importance of the top predictions and that the overall AUC value cannot reflect the top prediction results well, we conducted a consistency test to verify the top 100 prediction results. Based on the above training and independent testing dataset, the top 100 prediction results of plantMirP2, plantMirP and riceMirP were compared (Figure 4). The comparison results show the consistent effectiveness of plantMirP2. All in all, the results obtained above show that plantMirP2 is a promising predictor for plant pre-miRNAs.

**Figure 5:**
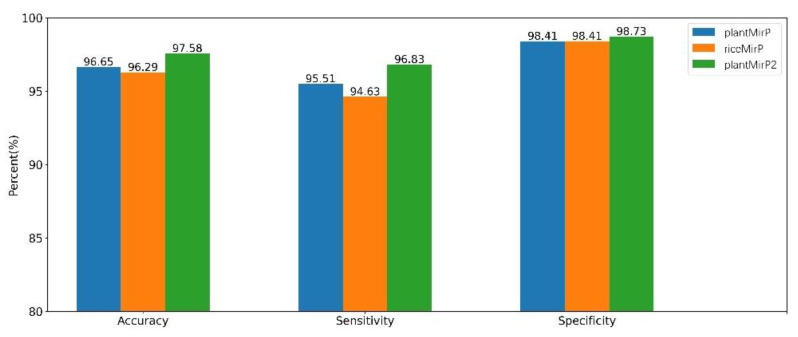
Based on the same training dataset and testing dataset, indicators of plantMirP, riceMirP and plantMirP2 were compared.

**Figure 6:**
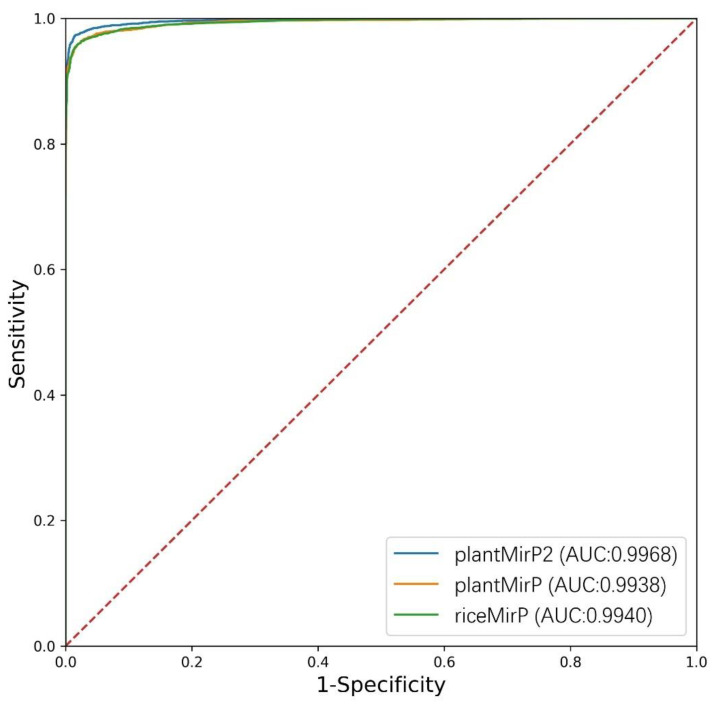
The ROC curves of plantMirP, riceMirP and plantMirP2.

PlantMirP is the first plant pre-miRNA tool with knowledge-based energy features calculated from distance-specific *k*-mer pair potentials. In plantMirP, knowledge-based energy features show very high discriminatory power. Recently, we further developed *k*-mer pair potentials, and put forward diverse knowledge-based energy features based on position-dependent *k*-mer pair potentials. Then, riceMirP was implemented specifically for rice pre-miRNAs. Numerous comparisons also demonstrate that riceMirP performs better than existing tools for other plant prediction. In order for performance improvement, we compared plantMirP2 with plantMirP and riceMirP based on the training and testing dataset of plantMirP2. The training dataset was used to train the prediction model for three tools, and the independent testing dataset was used for performance evaluation. The values of Ac, Se, Sp and AUC are displayed in Figure 5 and Figure 6. It is very clear that plantMirP2 is slightly superior to the other two tools.

### 3.2. Prediction for New Plant Pre-miRNAs in miRBase 22.1

**Figure 7:**
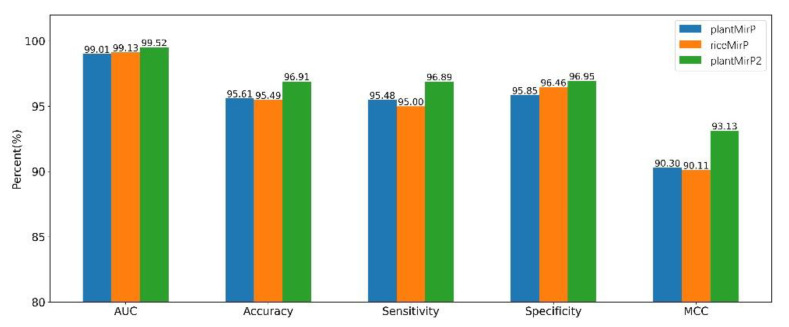
Based on the miRbase (release 21) training dataset and the miRbase (release 22.1) testing dataset, indicators of plantMirP, riceMirP and plantMirP2 were compared.

**Figure 8:**
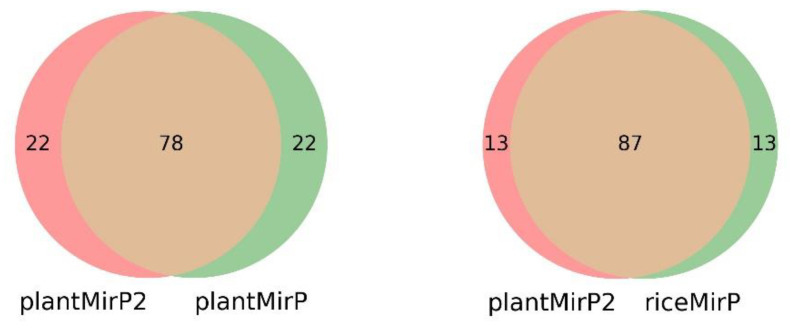
Venn diagram results for the top predictions of plantMirP2, plantMirP and riceMirP based on the miRbase (release 21) training dataset and the miRbase (release 22.1) testing dataset.

In order to further verify the performance of plantMirP2, 6944 pre-miRNA data in miRbase (release 21) and the corresponding 3472 negative data constructed through CDS sequences were used to construct the training dataset. The testing dataset consisted of 1639 new plant pre-miRNA data in miRbase (release 22.1) and 820 negative data. Based on the above datasets, we compared plantMirP2 with plantMirP and riceMirP. PlantMirP2 shows better performance than the other two tools (Figure 7). In addition, we also compared the top prediction results of the three tools. (Figure 8). All these results show a promising performance of plantMirP2 in plant pre-miRNA prediction.

### 3.3. Comparison with miPlantPreMat Based on Dataset of miPlantPreMat

**Figure 9:**
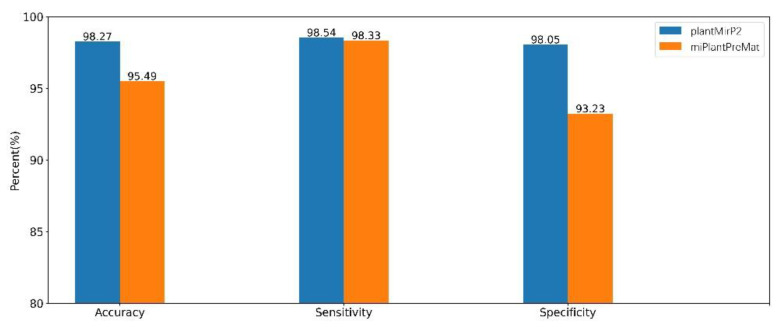
Based on the same dataset of miPlantPreMat, indicators of miPlantPreMat and plantMirP2 were compared.

MiPlantPreMat [19] is a representative computational program developed particularly for predicting plant pre-miRNAs. To avoid any bias in the dataset, the comparison with miPlantPreMat was carried out based on the training and testing dataset of miPlantPreMat. The negative dataset of miPlantPreMat was divided randomly into two parts: negData_training.txt and negData_testing.txt. The former was used for training and the latter was used for testing. We used the miPlantPreMat’s dataset (mirPlantPre19_single.txt and negData_training.txt) to train the prediction model of plantMirP2. Then, the miPlantPreMat dataset (mirPlantPre20_single.txt) and negative dataset (negData_testing.txt), which were considered to be the positive testing dataset and negative testing dataset, respectively, were directly submitted to plantMirP2 with retraining of the prediction model. Clearly, plantMirP2 shows a higher performance than miPlantPreMat (Figure 9).

### 3.4. Comparisons with PlantMiRNAPred, Triplet-SVM and MicroPred Based on Datasets of PlantMiRNAPred

**Figure 10:**
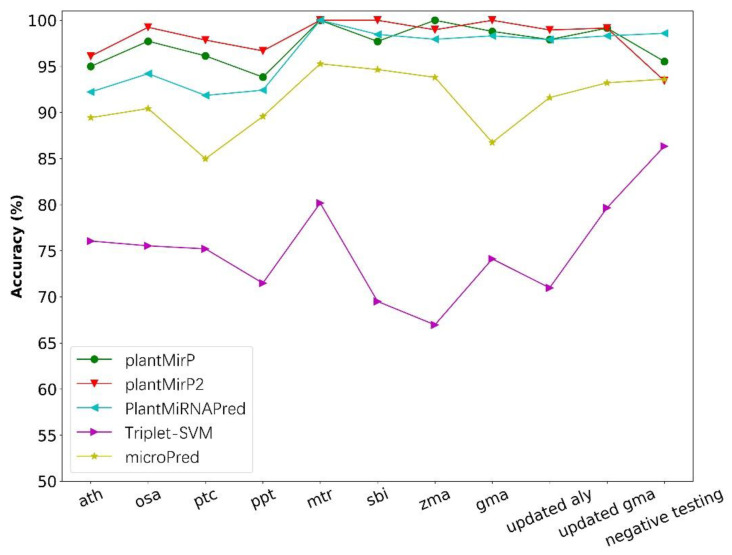
Based on the training and testing datasets from PlantMiRNAPred, the prediction accuracies of plantMirP, plantMirP2, PlantMiRNAPred, Triplet-SVM and microPred were compared.

The training dataset of PlantMiRNAPred, which included 980 real pre-miRNAs and 980 pseudo pre-miRNAs, was applied to training prediction models of plantMirP and plantMirP2. Then, the testing dataset of PlantMiRNAPred, which consisted of three parts, known pre-miRNAs from eight species, updated datasets and the negative testing dataset, was submitted directly into plantMirP and plantMirP2 for prediction with a freshly trained prediction model. Because PlantMiRNAPred is unavailable, the classification results of PlantMiRNAPred, triplet-SVM and microPred, which are reported in the article of PlantMiRNAPred, were used directly for comparisons with results obtained from our tools. It is very evident that plantMirP2 is superior to other tools in the great majority of datasets (Figure 10).

## 4. Conclusions

In order to predict miRNAs from NGS data, and further improve the prediction performance, speed and usability, we upgraded plantMirP to plantMirP2. The latest database data were used to build the prediction model. The optimized knowledge-based energy features and other effective features were widely combined to further improve the prediction accuracy. After optimizing the program and parameters of the prediction model, the prediction accuracy and running speed were also improved. Two prediction functions can be used through plantMirP2. The first function is pre-miRNA prediction. Users can use the established plant model based on the miRBase database or provide training data to establish a new model to predict the sequences. The second is miRNA prediction. Novel miRNAs can be obtained through NGS data with the help of genome files and ncRNAs files. By using a formatted GSM (Gene Expression Omnibus Sample) sequencing file, an *Arabidopsis thaliana* genome file and a related ncRNAs file for testing, 105 new high-precision miRNAs were successfully obtained [33]. The extensive comparisons with existing pre-miRNA prediction methods, such as plantMirP, riceMirP, miPlantPreMat, PlantMiRNAPred, triplet-SVM and microPred demonstrated that PlantMirP2 exhibits better performance. Taken together, plantMirP2 could be beneficial to relevant research. Furthermore, the easy-to-use webserver of plantMirP2 is provided at http://plantmi.top/ (accessed on 16 August 2021).

## Figures and Tables

**Table 1:** The differences in positive datasets of both tools.

Positive Dataset	Species	PlantMirP (Release 21)	PlantMirP2 (Release 22.1)
Training	Arabidopsis thaliana	325	326
	Glycine max	573	684
	Oryza sativa	592	604
	Physcomitrella patens	229	247
	Medicago truncatula	672	672
	Sorghum bicolor	205	205
	Arabidopsis lyrata	205	205
	Zea mays	166	168
	Solanum lycopersicum	77	112
Testing	Remaining plant species	3865	5323

**Table 2:** Full features used in plantMirP2.

NO.	Features	Description	Origin
1–34	Knowledge-based energy score1	Calculated using the position-specific contact potentials of 2-mer pairs.	riceMirP
35–39	Knowledge-based energy score2	Calculated using the distance-specific contact potentials of *k*-mer pairs (*k* = 1~5).	plantMirP
40–49	The ratio of unpaired nucleotide in sub-region 1–10	The secondary structure was divided into 10 parts and the ratio of unpaired nucleotide in each part was calculated.	plantMirP
50	the size of biggest bulge	The size of biggest bulge in secondary structure. A bugle contains at least three adjacent unpaired nucleotides.	plantMirP
51	n_stems/L	n_stems denotes the number of stems. A stem contains at least three continuous base pairs. L is the length of sequence.	plantMirP
52	n_loops/L	n_loops denotes the number of loops.	plantMirP
53	%(|G| + |C|)	(|G| + |C|)/L × 100. Here |X| denotes the number of base X in sequence.	miPred
54–69	%XY	|XY|/(L − 1) × 100. |XY| is number of dinucleotide XY in sequence.	miPred
70	dG = MFE/L	MFE is minimum of free energy of the secondary structure.	miPred
71	MFE1	(MFE/L)/%(|G| + |C|)	miPred
72	MFE2	(MFE/L)/n_stems	miPred
73	dP = tot_bases/L	tot_bases is number of base pairs in the secondary structure.	miPred
74	MFE3	(MFE/L)/n_loops	microPred
75–77	|X − Y|/L	|X − Y| is the number of base pairs, (X − Y)∈[(A − U), (G − C), (G − U)]	microPred
78–80	%(X − Y)/n_stems	%(X − Y) = |X − Y|/n_stems × 100	microPred
81	Avg_bp_stem1	tot_bases/n_stems	microPred
82	pb/nb	paired nucleotide/unpaired nucleotide	miRD
83	MCPN	Maximum of consecutive paired nucleotides.	ZmirP
84	n_bugles/L	n_bulges is the total number of bulges in the secondary structure.	ZmirP
85	Avg_bp_stem2	The ratio of number of base pairs to n_stems	ZmirP
86	MFE4	dG/tot_bases	ZmirP
87	MFE5	dG/n_bugles	ZmirP
88–167	*k*-mer features	*k*-mer features (*k* = 2 & 3).	milRP
168–193	Knowledge-based energy score3	Calculated using the distance-dependent *k*-mer pair potential (*k* = 1–3 and N_bins_ = 20).	milRP
